# Progression of retinitis pigmentosa on static perimetry, optical coherence tomography, and fundus autofluorescence

**DOI:** 10.1038/s41598-023-49338-0

**Published:** 2023-12-12

**Authors:** Yuhei Iga, Tomoko Hasegawa, Hanako Ohashi Ikeda, Yoshimichi Hirota, Manabu Miyata, Shogo Numa, Yuki Otsuka, Akitaka Tsujikawa

**Affiliations:** 1 Department of Ophthalmology and Visual Sciences, Kyoto University Graduate School of Medicine, 54 Shougoin Kawahara-cho, Sakyo-ku, Kyoto, 606-8507 Japan; 2 Japan Society for the Promotion of Science, Tokyo, Japan

**Keywords:** Retinal diseases, Hereditary eye disease

## Abstract

In retinitis pigmentosa (RP), photoreceptor degeneration leads to progressive visual field loss and visual impairment. Several therapeutic trials are ongoing aiming to establish effective treatments. Although functional evaluations are commonly used in clinical trials, residual ellipsoid zone (EZ) measurement on optical coherence tomography has been shown to be more sensitive to detect disease progression. Establishment of sensitive outcome measurement is essential to develop new therapeutic strategies. In the current study, we evaluated the progression rates of the disease in 76 eyes of 76 patients with RP, using the residual EZ length, ring-shaped macular hyperautofluorescent (AF), and visual field. Decrease rates measured by the residual EZ area and by the hyper-AF ring area were strongly positively correlated (*P* < 0.0001, r = 0.71). The reduction rates of the residual EZ length and hyper-AF ring radius were constant regardless of their baseline measurements. Faster annual reduction rates of the hyper-AF ring area or radius were significantly correlated with faster visual field progression (*P* = 0.03, r = 0.25 and *P* = 0.004, r = 0.33, respectively). These findings support the usage of morphological measurements such as EZ or hyper-AF ring measurements as outcome measurement for future clinical trials for RP.

## Introduction

Retinitis pigmentosa (RP) is a group of hereditary retinal degenerative diseases in which retinal photoreceptor cells are damaged, and visual impairment gradually progresses^1^. Several therapeutic ongoing trials aim to establish effective treatment strategies^2–4^. To monitor the progression of the disease, functional evaluations including visual acuity, visual field test, or electroretinography are commonly used in clinical trials^5–10^. However, fluctuations of the results in functional subjective evaluations, such as visual acuity or visual field test, are obstacles in the sensitive evaluation of the disease course or the efficacy of the therapies^11–13^. Residual ellipsoid zone (EZ) measurement on optical coherence tomography (OCT) has been shown to be more sensitive for detecting the progression of RP, with relatively high change rate with smaller inter-inspection fluctuations, compared to visual acuity or visual field test^13^. Morphological evaluations such as OCT or fundus autofluorescence (AF) have been reported to correlate well with functional evaluations^14–21^. In OCT measurements, longer residual EZ length has been shown to correlate well with better functions^14,15,22^. Macular AF images have been classified into three types: eyes with ring-shaped hyperautofluorescence, abnormal foveal hyperautofluorescence, and eyes without the above features^23^. In cases with ring-shaped hyperautofluorescence, longer ring diameter has been shown to correlate with longer residual EZ length and better retinal functions^16,22,24,25^. However, the expected relationship among the rates of progression by these different evaluation methods has not yet been clarified.

In the current study, we evaluated the progression rates of RP using the residual EZ length, ring-shaped macular hyperautofluorescent area, and visual field to determine the correlations among the progression rates with different measurements.

## Methods

### Participants

All methods in this retrospective longitudinal study were carried out in accordance with the Declaration of Helsinki and the Ethical Guidelines for Medical and Health Research Involving Human Subjects by Ministry of Health, Labour and Welfare of Japan. This study was approved by the Institutional Review Board and Ethics Committee of the Kyoto University Graduate School of Medicine. Due to the retrospective design of this study, the need for written informed consent was waived by the Institutional Review Board and Ethics Committee of the Kyoto University Graduate School of Medicine.

The clinical records of patients with RP who visited the Kyoto University Hospital between May 2011 and May 2021 were reviewed. RP was diagnosed based on the clinical history and findings of comprehensive ophthalmologic examinations, including fundus examination, visual acuity measurements, visual field tests, OCT imaging, wide-field fundus imaging, and electroretinography. Eyes that were monitored for more than 1.5 years by OCT (using Spectralis® HRA + OCT system [Heidelberg Engineering, Heidelberg, Germany]), wide field fundus autofluorescence (AF) imaging (Optos, [Optos, Inc., MA, USA]), and by four or more automated perimetry tests (except for the first test^26,27^) (10-2 Swedish interactive threshold algorithm standard program; Humphrey Visual Field Analyzer, [Carl Zeiss Meditec, Inc. software, CA, USA], referred to as HFA 10-2 hereafter) were candidates for inclusion in the study. Macular AF images were classified as described in a previous report^23^ as follows: eyes with a ring-shaped hyperautofluorescent area around the fovea (referred to as hyper-AF ring hereafter), eyes with abnormal foveal hyperautofluorescence, and eyes without the above findings. Eyes with hyper-AF rings were included in this study. Eyes with other retinal or optic nerve diseases, a history of intraocular surgeries other than cataract surgery, intraocular surgeries during the observation period, and eyes whose EZ length on OCT images or hyper ring area on AF were unmeasurable at the beginning of the observation period were excluded from the study. When both eyes of a patient were eligible, only the eye with a milder visual field defect at the initial HFA10-2 (decided by the mean deviation [MD] value) was included.

### Acquisition and analysis of visual field tests, OCT, and AF

For the visual field test analysis, the MD value of HFA 10-2 was analyzed. HFA tests with unreliable outcomes, such as fixation loss of > 20%, a false-positive rate of > 15%, or false-negative rate of > 33%, were excluded. The first HFA 10–2 examinations were excluded, accounting for subject-learning effects^26,27^.

**Figure 1:**
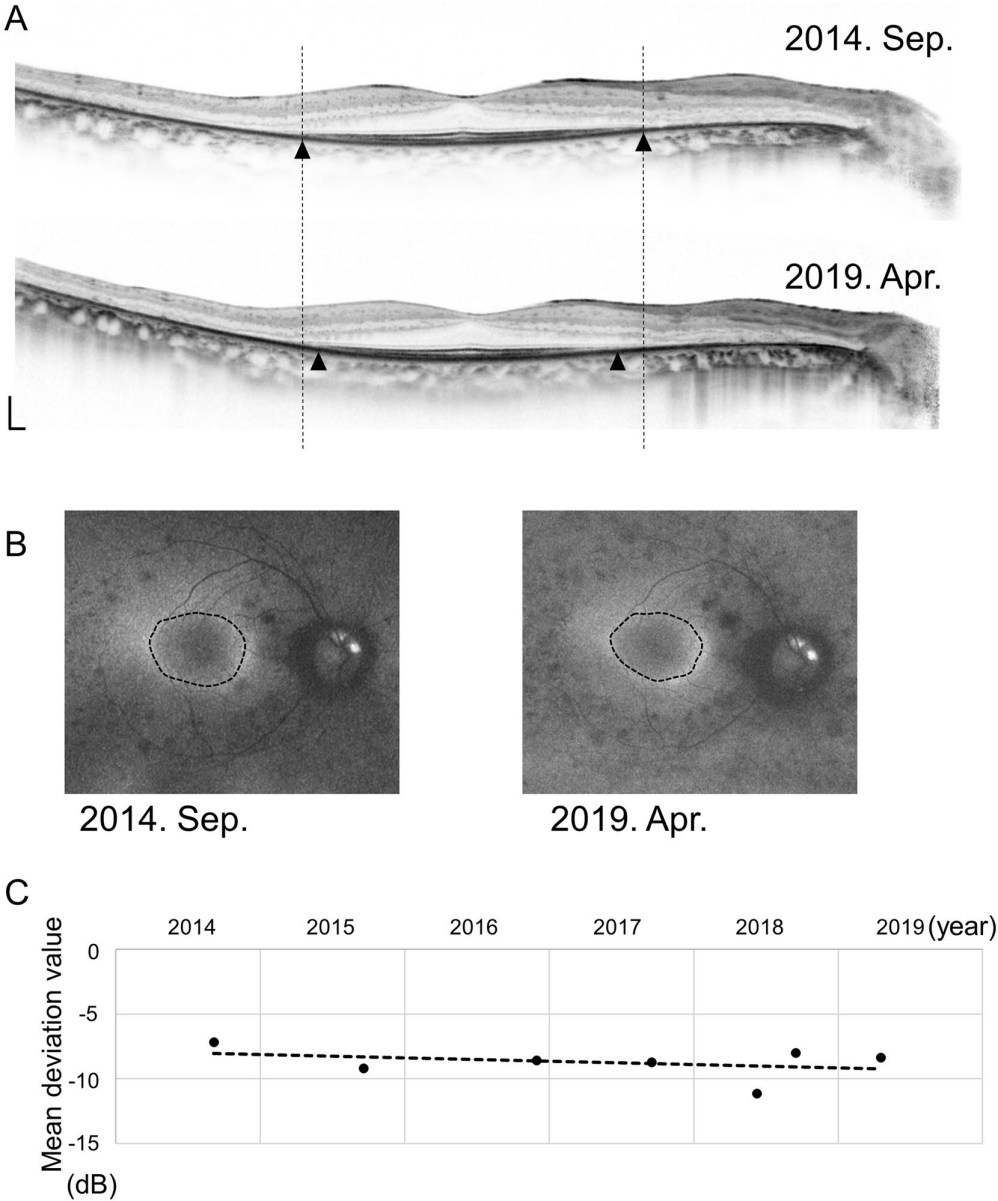
Images of the right eye of a 38-year-old man with retinitis pigmentosa. (**A**) Residual ellipsoid zone (EZ) length on spectral-domain optical coherence tomography (SD-OCT) images over time. The EZ length is measured as the distance between the black arrowheads. The dotted lines show the end of the EZ length at baseline to clarify the changes. Scale bar: 200 μm. (**B**) Autofluorescence (AF) images showing ring-shaped hyper-AF (hyper-AF ring) over time. The dotted lines show the center of the inner and outer edges of the hyper-AF ring. (**C**) Mean deviation (MD) values on automated perimetry (10-2 Swedish interactive threshold algorithm standard program; Humphrey Visual Field Analyzer) over time. The dotted line shows the approximate straight line using linear regression analysis with the least-squares method.

The macula was scanned using OCT. The residual EZ length was measured on horizontal and vertical 30° scans crossing the fovea using the built-in software in the Heidelberg Engineering OCT system (Fig. 1A). The average of the horizontal and vertical lengths was calculated and used as the residual EZ length for the analysis^13^. Additionally, the measured values of the horizontal and vertical residual EZ lengths were used to calculate the residual EZ area approximated as an ellipse, and the area was calculated as π (horizontal length)(vertical length)/4.

The area of the hyper-AF ring was measured as the area within the centerline tracing the center of the inner and outer edges of the hyper-AF ring using a built-in software in Optos Viewer (Optos, Inc. MA, USA) (Fig. 1B). The measured area was used to calculate the virtual radius as the square root of [(area)/π].

For OCT and autofluorescence imaging, only the images at the beginning and end of the observation period were utilized for analysis.

### Statistical analysis

The annual progress rate was evaluated by dividing the change value during the observation period by the observation period length for the residual EZ length and EZ area on OCT and the hyper-AF ring area and AF ring radius on fundus AF. Annual % change was calculated by dividing the annual progress rate by the baseline measured values. To evaluate the annual progress rate of the visual field, the MD slope was calculated using linear regression analysis with the least-squares method (Fig. 1C).

Correlations between the baseline values and annual progress rates for each evaluation method were evaluated using Pearson's linear approximation.

Annual progress rates of residual EZ length and the AF ring radius were compared between eyes with baseline EZ length values ≥ 3000 μm and < 3000 μm and between eyes with baseline AF ring radius values ≥ 1500 μm and < 1500 μm using the unpaired t-test. The MD slope was compared among eyes with a baseline of < − 25 dB, − 5 ~ − 25 dB, and > − 5 dB using analysis of variance. For the comparison of these annual progress rates with the baseline, outliers were defined as eyes with annual progress rates out of the range between “(the first quartile) − 1.5 (interquartile range)” and “(the third quartile) + 1.5 (interquartile range).” Statistical analysis was performed using JMP Pro software (v. 17; SAS Institute Japan Ltd., Tokyo, Japan). Data are presented as means ± standard deviations, where applicable.

## Results

Of the 602 candidate eyes, 170 eyes (28.2%) had hyper ring type AF. Of these, 12 eyes whose EZ length on OCT images or hyper ring area on AF were unmeasurable at the beginning of the observation period were excluded; and 8 eyes that met other exclusion criteria such as intraocular surgeries during the observation period or a history of intraocular surgeries other than cataract surgeries, were excluded. Only the eye with a milder visual field defect at the initial HFA10-2 was included when both eyes of the patient were eligible. Finally, 76 eyes from 76 patients were included in the analysis.

**Figure 2:**
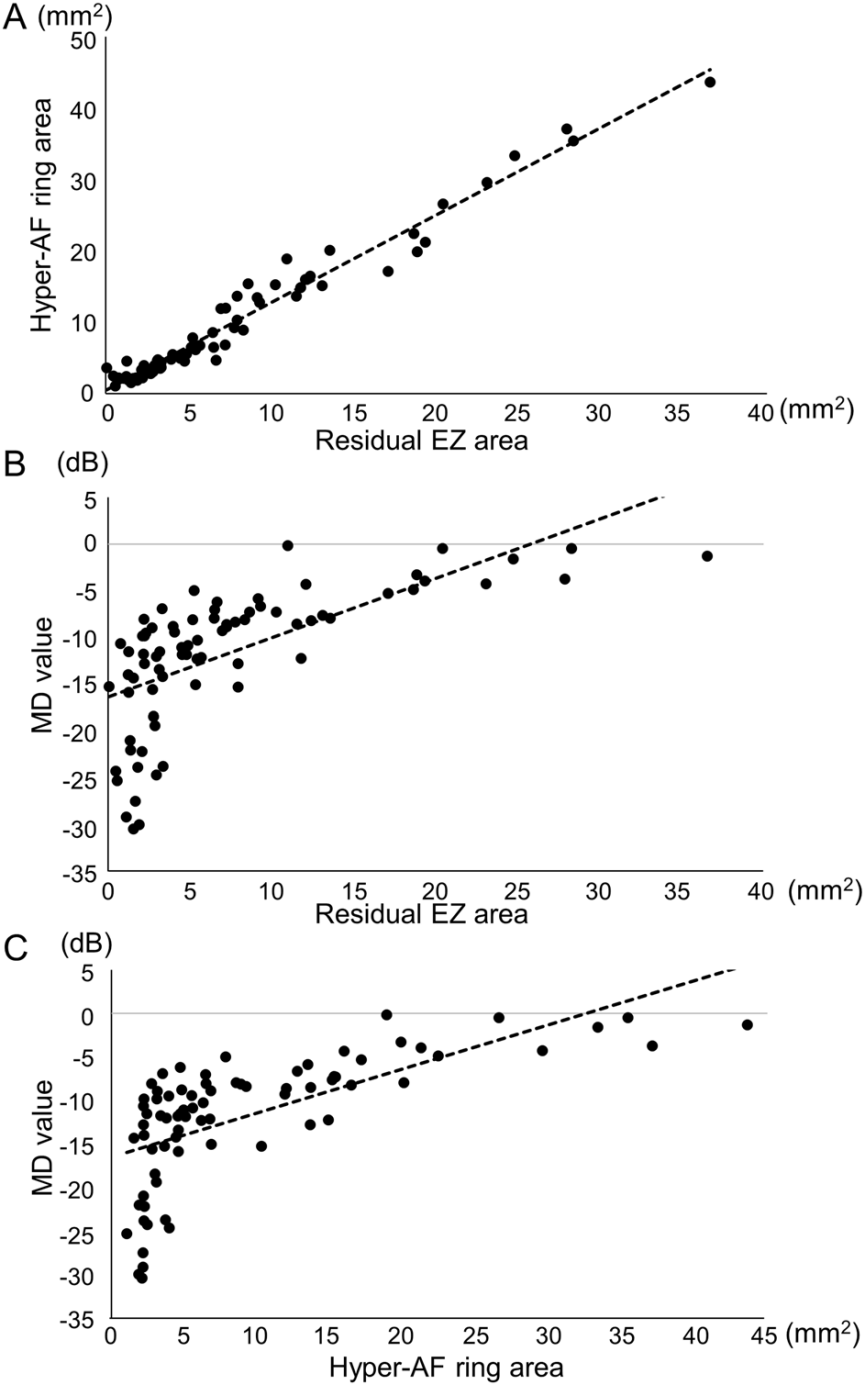
Correlations between the different baseline measurements. (**A**) Residual ellipsoid zone (EZ) area and the hyperautofluorescent ring (hyper-AF ring) area show a positive correlation (*P* < 0.0001, r = 0.98, Pearson's linear approximation). (**B**) Residual EZ area shows a positive correlation with mean deviation (MD) value (*P* < 0.0001, r = 0.65). (**C**) Hyper-AF ring area shows a positive correlation with MD value (*P* < 0.0001, r = 0.65). The dotted lines show the approximate straight line using linear regression analysis with the least-squares method.

The baseline characteristics of the patients are summarized in Table 1. The mean age of the patients was 44.0 ± 16.3 years at baseline. The MD was − 11.7 ± 7.2 dB, residual EZ area was 7.3 ± 7.4 mm^2^, residual EZ length was 2746 ± 1386 μm, hyper-AF ring area was 9.4 ± 9.3 mm^2^, and hyper-AF ring radius was 1567 ± 748 μm, at baseline (Table 1). Presumed causative genes were confirmed in 55.3% of the patients (42 patients); *EYS* accounted for 23.7% (18 patients), *PDE6B* 6.6% (5 patients), *RHO* 5.3% (4 patients) and *USH2A* 5.3% (4 patients) (Table 1). The baseline residual EZ length and area were strongly positively correlated with the hyper-AF ring radius and area (*P* < 0.0001, r = 0.97 and *P* < 0.0001, r = 0.98, respectively) (Fig. 2A, Supplementary Fig. S1A). The MD value was also strongly positively correlated with the residual EZ length and area (*P* < 0.0001, r = 0.72 and *P* < 0.0001, r = 0.65, respectively) (Fig. 2B, Supplementary Fig. S1B) and with the hyper-AF ring radius and area at baseline (*P* < 0.0001, r = 0.71 and *P* < 0.0001, r = 0.65, respectively) (Fig. 2C, Supplementary Fig. S1C).

**Table 1:** Baseline clinical characteristics of the participants.

Sex (female/male)	47/29
Age (years)	44.0 ± 16.3
MD value (dB)	− 11.7 ± 7.2
Residual EZ area (mm^2^)	7.3 ± 7.4
Residual EZ length (µm)	2746 ± 1386
Hyper-AF ring area (mm^2^)	9.4 ± 9.3
Hyper-AF ring radius (µm)	1567 ± 748
Presumed causative gene, n patients (%)	
Confirmed cases of causative genes	42 (55.3%)
*EYS*	18 (23.7%)
*PED6B*	5 (6.6%)
*RHO*	4 (5.3%)
*USH2A*	4 (5.3%)
*MAK*	2 (2.6%)
*RP1L1*	2 (2.6%)
*FSCN2*	2 (2.6%)
* PRPH2*	1 (1.3%)
* PRPF6*	1 (1.3%)
*PRPF8*	1 (1.3%)
*PRPF31*	1 (1.3%)
*TOPORS*	1 (1.3%)
Unclear	30 (51.3%)
Not determined	4 (5.3%)

Data are presented as means ± standard deviations or a number.

MD: mean deviation value on automated perimetry (10-2).

EZ: ellipsoid zone on optical coherence tomography.

hyper-AF ring: ring-shaped hyperautofluorescent findings around the fovea on wide-field fundus autofluorescence imaging.

### The annual progression rates and baselines

Included patients had HFA 10-2 for 5.7 ± 1.4 times over 5.8 ± 2.3 years of the observation period. The overall annual change rate was − 0.30 ± 0.52 dB/year for MD, − 0.29 ± 0.42 mm^2^/year (− 5.8 ± 5.1%) for the residual EZ area, − 73.6 ± 68.7 µm/year (− 3.3 ± 3.0%) for the residual EZ length, − 0.38 ± 0.43 mm^2^ /year (− 5.2 ± 5.0%) for the hyper-AF ring area, and − 40.6 ± 37.4 µm/year (− 3.0 ± 3.1%) for the hyper-AF ring radius (Table 2).

**Table 2:** Annual change rate of the participants with different parameters.

	Annual change
MD (MD slope, dB/year)	− 0.30 ± 0.52
Residual EZ area (mm^2^/year, % change/year)	− 0.29 ± 0.42 (− 5.8 ± 5.1%)
Residual EZ length (µm/year, % change/year)	− 73.6 ± 68.7 (− 3.3 ± 3.0%)
Hyper-AF ring area (mm^2^/year, % change/year)	− 0.38 ± 0.43 (− 5.2 ± 5.0%)
Hyper-AF ring radius (µm/year, % change/year)	− 40.6 ± 37.4 (− 3.0 ± 3.1%)

Data are presented as means ± standard deviations.

MD: mean deviation value on automated perimetry (10–2).

EZ: ellipsoid zone on optical coherence tomography.

hyper-AF ring: ring-shaped hyperautofluorescent findings around the fovea on wide-field fundus autofluorescence imaging.

**Figure 3:**
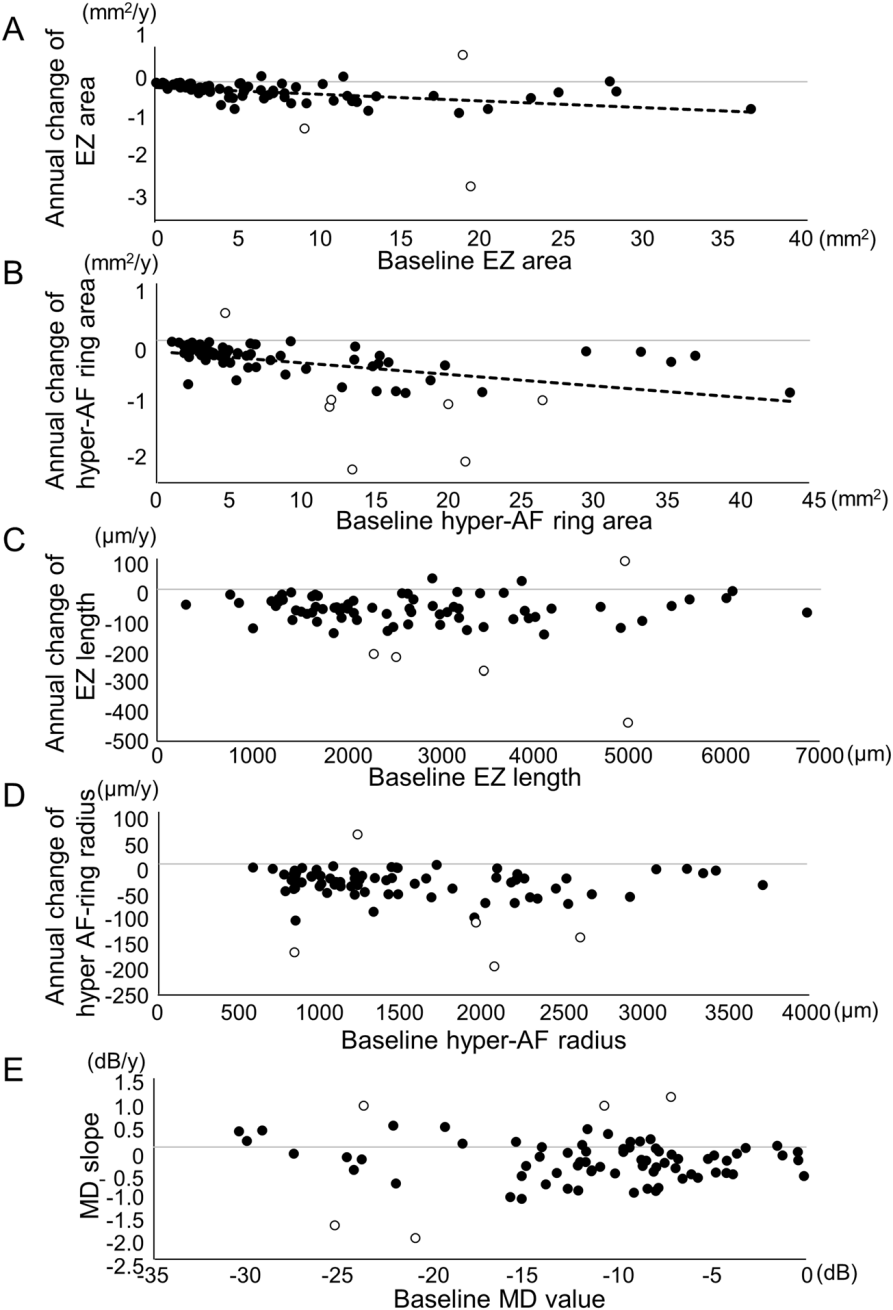
Correlations between the annual change rate and baseline measurements. (**A**) Larger baseline residual ellipsoid zone (EZ) area correlated with a faster annual reduction of the residual EZ area (*P* = 0.003, r = − 0.34, Pearson’s linear approximation). (**B**) Larger baseline hyperautofluorescent ring (hyper-AF ring) area correlated with faster annual reduction (*P* < 0.0001, r = − 0.44). (**C**) The baseline residual EZ length does not show a significant correlation with the annual reduction rate of EZ length (*P* = 0.40, r = − 0.10). (**D**) The baseline hyper-AF ring radius does not show a significant correlation with the annual reduction rate of the hyper-AF ring radius (*P* = 0.27, r = − 0.13). (**E**) There is no significant correlation between the baseline MD and MD slope (*P* = 0.61, r = − 0.06). The dotted lines in (**A**) and (**B**) show the approximate straight line using linear regression analysis with the least-squares method. Open dots show the outliers for annual change rates, which are defined as out of range between “(the first quartile) − 1.5 (interquartile)” and “(the third quartile) + 1.5 (interquartile).”

The larger the baseline residual EZ area, the faster the area was reduced (Fig. 3A, *P* = 0.003, r = − 0.34). Similarly, the larger the baseline hyper-AF ring area, the faster the area was reduced (Fig. 3B, *P* < 0.0001, r = − 0.44). When the residual EZ length or the radius of the hyper-AF ring was used for the analysis, there was no significant correlation between the baseline and change rate (Fig. 3C and D; *P* = 0.40, r = − 0.10 for EZ length;* P* = 0.27, r = − 0.13 for hyper-AF ring radius). The annual changes in residual EZ length were − 82.7 μm/year and − 68.9 μm/year, with a baseline length of  ≥ 3000 μm and < 3000 μm, respectively (*P* = 0.41). Of the five outliers of the annual changes in the EZ length, four were in the negative direction (Fig. 3C). The annual changes in the radius of the hyper-AF ring were − 50.3 μm/year and − 36.1 μm/year, with a baseline radius of  ≥ 1500 μm and < 1500 μm, respectively (*P* = 0.12). Of the five outliers of the annual changes in the hyper-AF ring radius, four were in the negative direction (Fig. 3D).

Similarly, in eyes with the causative gene *EYS*, larger baseline areas of the EZ and hyper-AF ring corresponded to a trend of faster reduction in these areas (Supplementary Fig. S2).

There was no significant correlation between the baseline MD and MD slope (Fig. 3E, *P* = 0.61). The MD slope was − 0.20 ± 0.87 in eyes with a baseline MD of < − 25 dB (N = 5 eyes), − 0.31 ± 0.54 in eyes with a baseline MD within the range of − 5 ~ − 25 dB (N = 59 eyes), and − 0.30 ± 0.24 in eyes with a baseline MD of  > − 5 dB (N = 12 eyes, *P* = 0.91, among the three groups).

### Correlations between progression rates evaluated by different parameters

**Figure 4:**
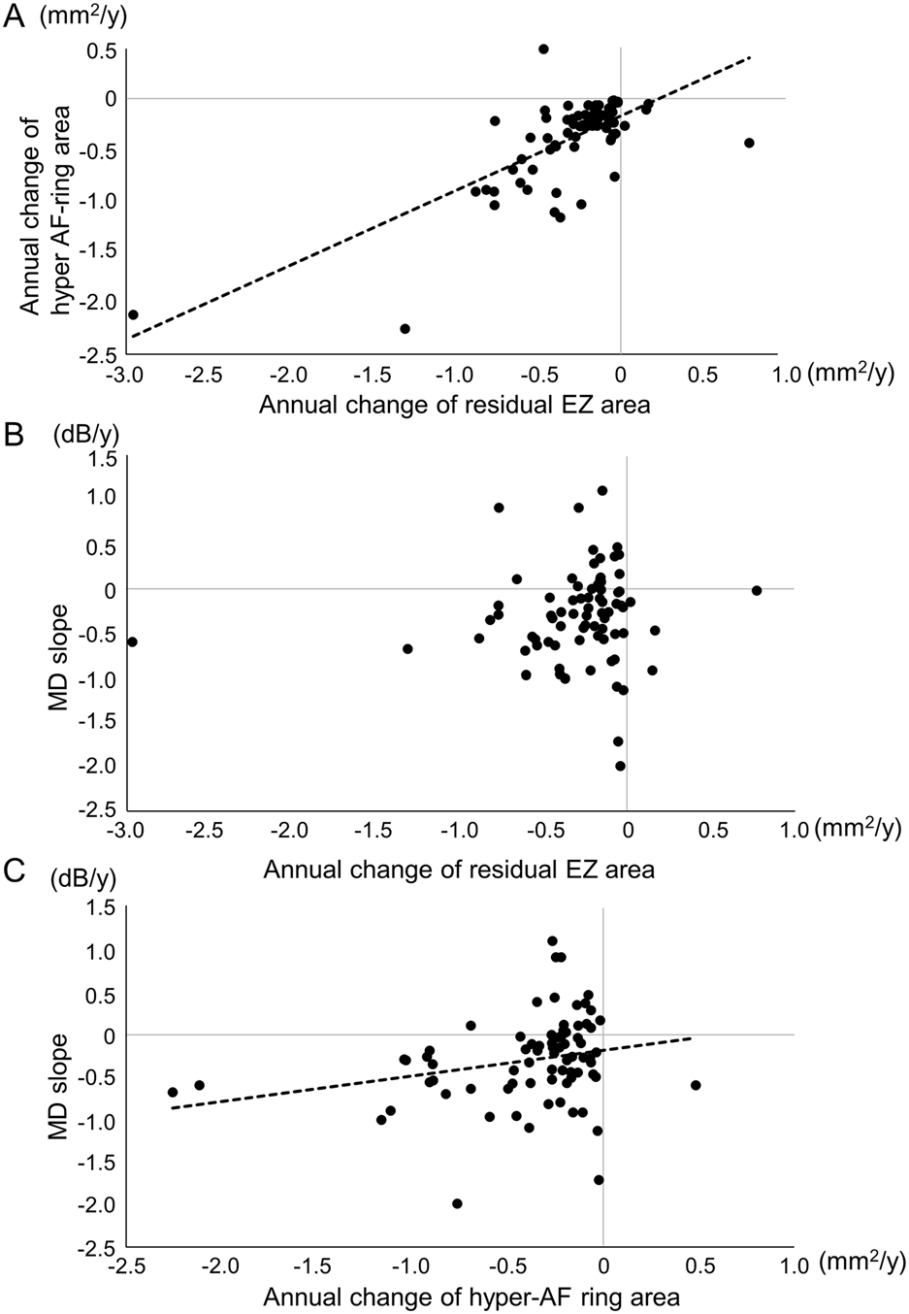
Correlations between annual change rates evaluated by different parameters. (**A**) The annual decrease rates of the residual EZ and hyper-AF areas are positively correlated (*P* < 0.0001, r = 0.71). (**B**) The annual change rate of the residual EZ area does not show a significant correlation with the MD slope (*P* = 0.49, r = 0.080). (**C**) Faster annual reduction rates of the hyper-AF ring area are significantly correlated with a faster MD slope (*P* = 0.03, r = 0.25). The dotted lines in (**A**) and (**C**) show the approximate straight line using linear regression analysis with the least-squares method.

Decrease rates of the residual EZ and hyper-AF ring areas were strongly and positively correlated (Fig. 4A, *P* < 0.0001, r = 0.71). Decrease rates of the residual EZ length and hyper-AF ring radius were also positively correlated (*P* < 0.0001, r = 0.48).

No significant correlation was detected between the rate of change in the residual EZ area nor the residual EZ length and the MD slope (Fig. 4B, *P* = 0.49 and *P* = 1.00, respectively). In contrast, faster annual reduction rates of the hyper-AF ring area and hyper-AF ring radius were significantly correlated with faster visual field progression (Fig. 4C, *P* = 0.03, r = 0.25 and* P* = 0.004, r = 0.33, respectively). Similarly, in eyes with causative gene *EYS*, positive correlations were observed between the rates of decrease in the residual EZ and hyper-AF ring areas, as well as between decrease rates of the hyper-AF ring area and MD slope (Supplementary Fig. S3, *P* = 0.0002, r = 0.78 and *P* = 0.045, r = 0.48, respectively).

## Discussion

The disease course of RP is monitored by functional evaluations, including visual acuity, perimetry, and electroretinography, and by morphological evaluations, including OCT and fundus AF. In clinical trials for RP, functional evaluations such as: visual acuity or the visual field test are commonly utilized. However, fluctuations in these results pose challenges in sensitively detecting disease progression or the efficacy of therapies^13^, compounding the already difficult task due to the substantial individual variability in the disease course among patients with RP. Sensitive and sufficient monitoring of disease progression and the effectiveness of therapies is indispensable in clinical trials. Although a longer residual EZ length or longer ring diameter of the hyper-AF ring has been shown to correlate well with better retinal functions^14–16,22,24,25^, the relationship between the rates of progression has not yet been clarified.

In the current study, we evaluated the disease progression in RP with visual field test, OCT imaging, and fundus AF imaging. We found that the reduction rates of the EZ and hyper-AF ring areas were significantly positively correlated with each other. Moreover, the rate of visual field progression was significantly and positively correlated with the rate of reduction of the hyper-AF ring area; the faster the visual field progression, the faster the reduction of the hyper-AF ring area (Fig. 4). These results will support the use of morphological evaluations, such as EZ or AF ring measurements, in future clinical trials. Nevertherless, these morphological evaluations also possess several limitations. Firstly, they cannot mitigate the substantial standard deviations caused by individual variations among patients. Secondly, similar to functional evaluations, they exhibit a floor effect, as indicated in Fig. 3. Thirdly, concerning the AF assessment, various types of AF patterns may not be compatible with one another. Essentially, utilizing a specific AF pattern, such as the hyper-AF ring pattern in our current study, will result in the exclusion of a significant number of patients with different AF patterns.

At baseline, the MD, residual EZ area on OCT measurement, and hyper-AF ring area were strongly correlated with each other, as previously reported^24,25,28^. In addition, we examined the possible relationships between the baseline and progression rates for each parameter. Regarding the visual field, the MD change rate differences among different baselines were not statistically significant in the current study (Fig. 3E) but were not contradictory to a previous report that showed a faster decrease rate in eyes with baseline MD values between − 5 and − 25 dB^29^.

The change rate of the AF ring and EZ length has been reported to be faster in eyes in which the baseline AF ring diameter or the EZ length is larger than 3000 μm^25,28^. In the current study, the annual changes in the residual EZ length and hyper-AF ring radius did not correlate significantly with their baselines and showed almost constant annual change rates regardless of their baselines (Fig. 3C and D). RP is a highly variable disorder, and there is great variability in disease severity or disease progression rate^1^. There were outliers with extremely fast annual change rates in the current study (Fig. 3), which can affect the average annual change rates. Additionally, patients with slower annual changes may not notice their symptoms at the early stages of the disease and may not visit hospitals unless their disease progresses to a certain level, which means that patients with a slower annual change rate might only be included when they are in more advanced stages in hospital-based studies such as the current study. To clarify whether the annual progression rate changes depending on the baseline, or as the disease progresses, a longer observation of the same patient cohort is needed, although intraocular surgeries or other complications might be obstacles in studies with longer observation periods. As for the calculated areas as opposed to length, there were significant correlations between the baseline and progression rate; the larger the baseline AF ring area or the residual EZ area, the faster the decrease. It is reasonable that the change rate of the AF ring or the residual EZ area is faster with larger baseline when the progression rate as a function of length (diameter or radius) is constant.

The borderline areas where the progression occurred in most of the eyes included in the current study were located where the density of rods is higher on the more outward side, and the cones are way less than the rods^30^. Considering together with that the change was faster with larger baseline EZ or hyper-AF ring areas, it is suggested that photoreceptors were damaged faster in locations with higher densities. Apoptosis, autophagy, and necroptosis have been indicated in photoreceptor cell death in inherited retinal degeneration^31–33^. Damage-associated molecular patterns (DAMPs) can be released during cell death, such as necrosis, necroptosis, apoptosis, or autophagy, and can cause inflammation and tissue injury^34–37^. Faster photoreceptor degeneration in areas with higher photoreceptor densities may indicate that photoreceptor cell death in places with higher photoreceptor density accelerates cell damage through DAMPs.

This study has several limitations. First, this study included eyes that were monitored for a minimum period of more than 1.5 years. Although the included eyes underwent four or more automated perimetry tests to increase the reliability of the analysis of visual field progression, 1.5 years of observation might not be sufficiently long to effectively evaluate the visual field progression rate. Additionally, as mentioned above, to clarify whether the annual rate of change would change depending on the baseline or as the disease progresses, longer observations of the same population are needed. Second, eyes with earlier and more advanced stages were analyzed together to determine the correlations between progression rates among different parameters. The rate of progress may vary among different parameters depending on the disease stages^29^. The number of eyes with very early or advanced stages included in the current study was small, partly because patients with very early stages tended to visit hospitals less frequently, and eyes with severe advanced stages were excluded due to unmeasurable EZ length at baseline. Third, owing to limited case numbers per causative gene, this study collectively analyzed cases with different genes. Although a consistent pattern emerged for cases with the *EYS* gene, a possibility exists that the genotype affects phenotype variation across parameters^38–42^. Fourth, EZ length was analyzed as a measurable maximum length despite discontinuities in some cases. It might be more accurate if we could account for the discontinuity, although accurate measurement of the length of each of the discontinuous fragment was challenging when the EZ was damaged. Additionally, we used horizontal and vertical cross-sections rather than volume scans for the EZ analysis to include as many cases as possible, as most candidate patients underwent cross scans rather than volume scans with OCT. Reduction rates of the EZ and visual field did not show significant correlation in the current study. Using volume scans, which directly measure the intact EZ area, might be more suitable for comparison. Fifth, we used mean deviation as the visual field measurement in this study. Although mean deviation was chosen because of its widespread use in clinical settings, other measurements such as total point score, sum of visual sensitivities of all measured locations, might be more suitable for comparison with morphological areal measurement. Finally, this study only analyzed eyes with a ring-shaped hyper-AF area around the fovea. The exclusion of eyes with other AF patterns consequently eliminated a considerable proportion of eyes; only 28.2% of the eyes showed a hyper-AF ring pattern of AF in the current study. Progression with other AF patterns remains to be elucidated.

In conclusion, the annual rate of change of the hyper-AF ring area was significantly positively correlated with that of the visual field (MD) and remnant EZ length area. These findings will form the basis for the use of these morphological evaluations, which are sensitive parameters with smaller fluctuations, in future clinical trials.

### Supplementary Information

Supplementary Figures.

## Data Availability

All data generated or analyzed during this study are included in this published article and its Supplementary Information files.
