# Supplementary material for: Progression of retinitis pigmentosa on static perimetry, optical coherence tomography, and fundus autofluorescence
Source: Sci Rep. 2023 Dec 12;13:22040. doi: 10.1038/s41598-023-49338-0 (PMC10716373; doi:10.1038/s41598-023-49338-0)
Supplement: Supplementary file 1 — Supplementary Figures. [file 41598_2023_49338_MOESM1_ESM.docx]

**Supplementary information**

**Progression of retinitis pigmentosa on static perimetry, optical coherence tomography, and fundus autofluorescence**

Yuhei Iga, Tomoko Hasegawa, Hanako Ohashi Ikeda, Yoshimichi Hirota, Manabu Miyata, Shogo Numa, Yuki Otsuka, Akitaka Tsujikawa

**
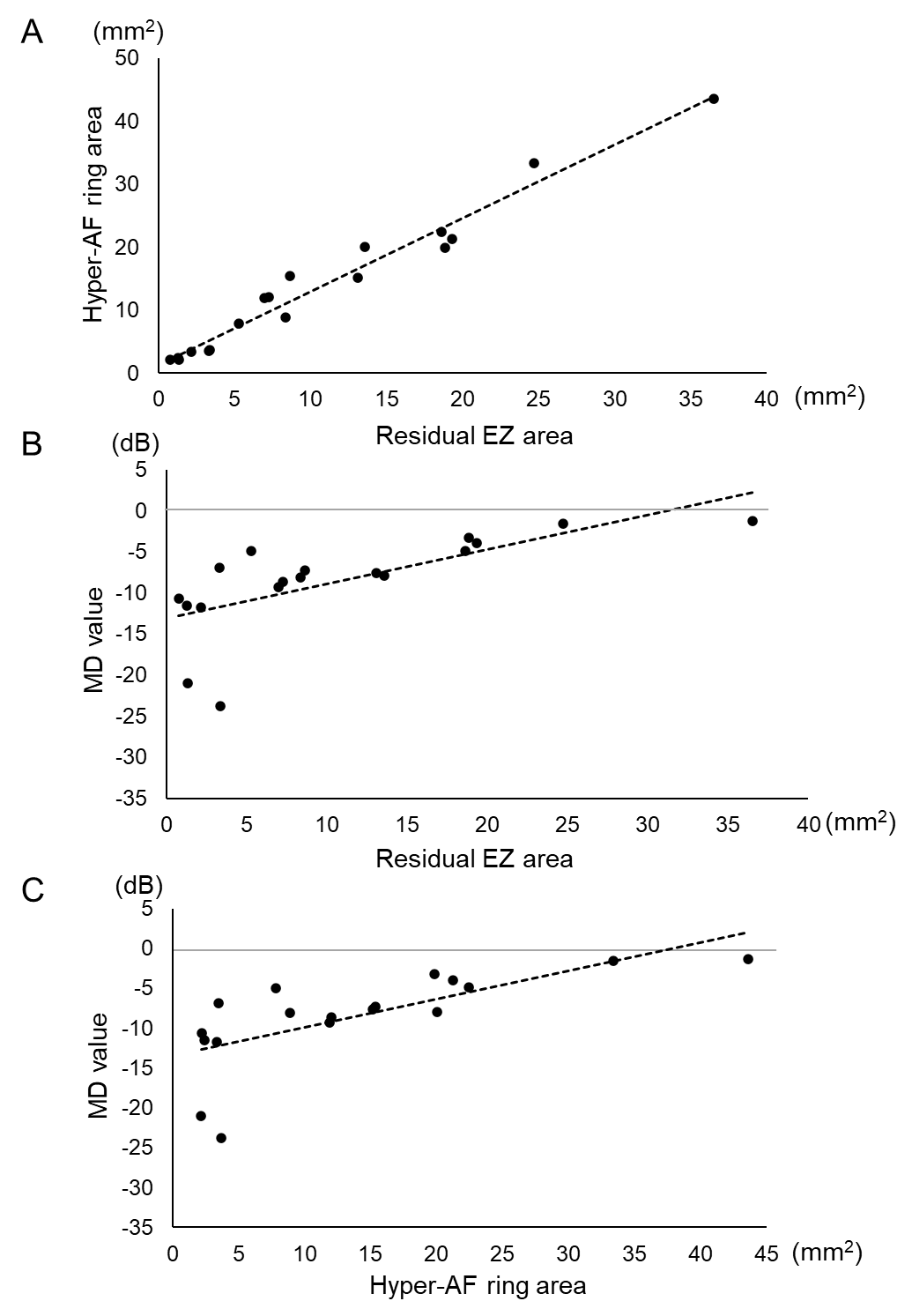
**

**Supplementary Figure S1. Correlations between different baseline measurements in eyes with causative *EYS* gene. (A)** Residual ellipsoid zone (EZ) area and the hyper-autofluorescent ring (hyper-AF ring) area exhibit a positive correlation (*P* < 0.0001, r = 0.98, Pearson's linear approximation). **(B)** Residual EZ area displays a positive correlation with mean deviation (MD) value (*P* = 0.0015, r = 0.69). **(C)** Hyper-AF ring area shows a positive correlation with MD value (*P* = 0.0013, r = 0.70). The dotted lines represent the approximate straight line using linear regression analysis with the least-squares method.

**
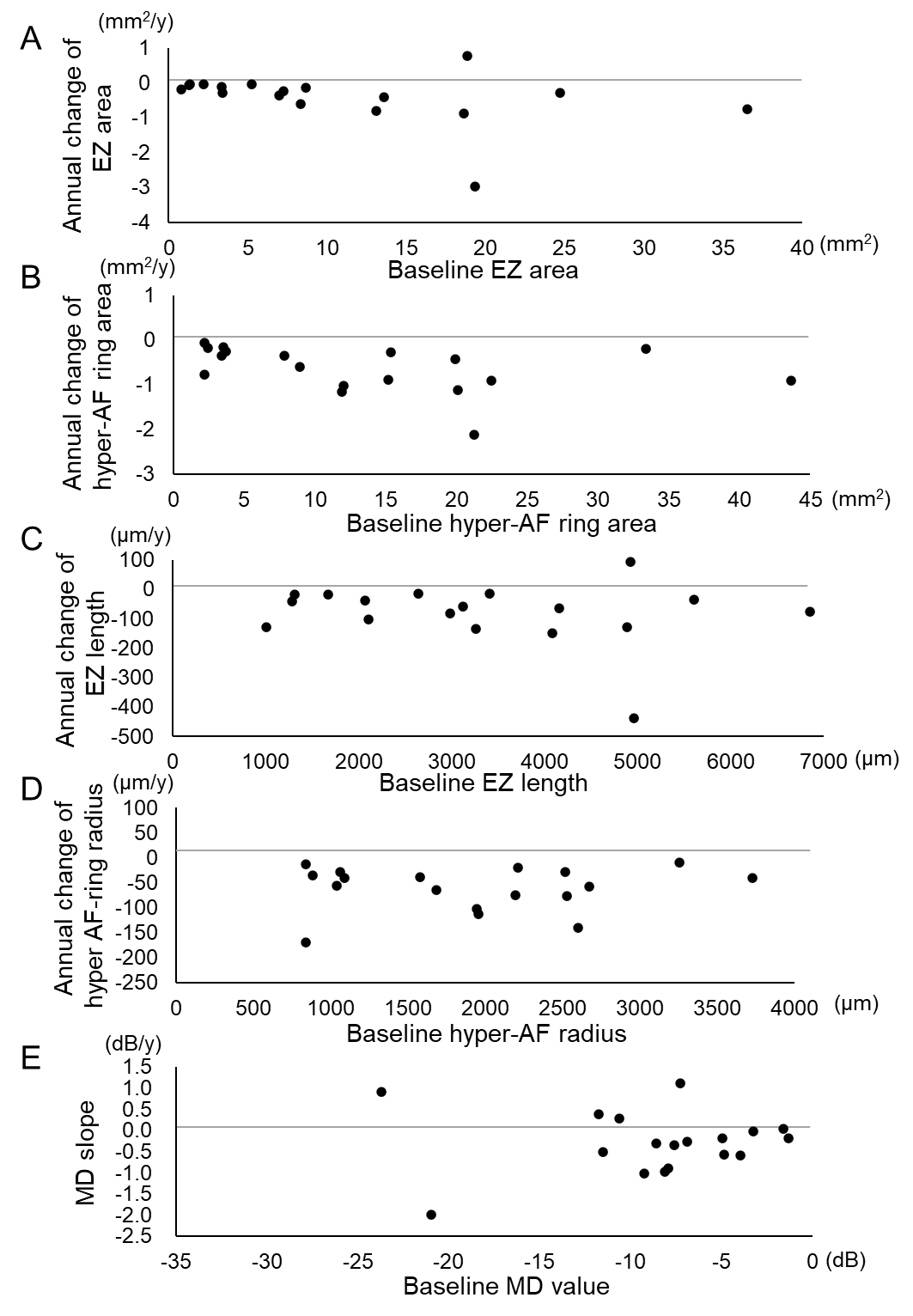
**

**Supplementary Figure S2.** **Correlations between the annual change rate and baseline measurements in eyes with causative *EYS* gene.** **(A)** A larger baseline residual ellipsoid zone (EZ) area showed a tendency to be correlated with a faster annual reduction of the residual EZ area (*P* = 0.16, r = -0.35, Pearson’s linear approximation). **(B)** Larger baseline hyper-autofluorescent ring (hyper-AF ring) area exhibited a tendency to be correlated with a faster annual reduction (*P* = 0.14, r = -0.36). **(C)** The baseline residual EZ length does not display a significant correlation with the annual reduction rate of EZ length (*P* = 0.46, r = -0.19). **(D)** The baseline hyper-AF ring radius does not exhibit a significant correlation with the annual reduction rate of the hyper-AF ring radius (*P* = 0.77, r = -0.07). **(E)** No significant correlation between the baseline MD and MD slope (*P* = 0.83, r = -0.06).


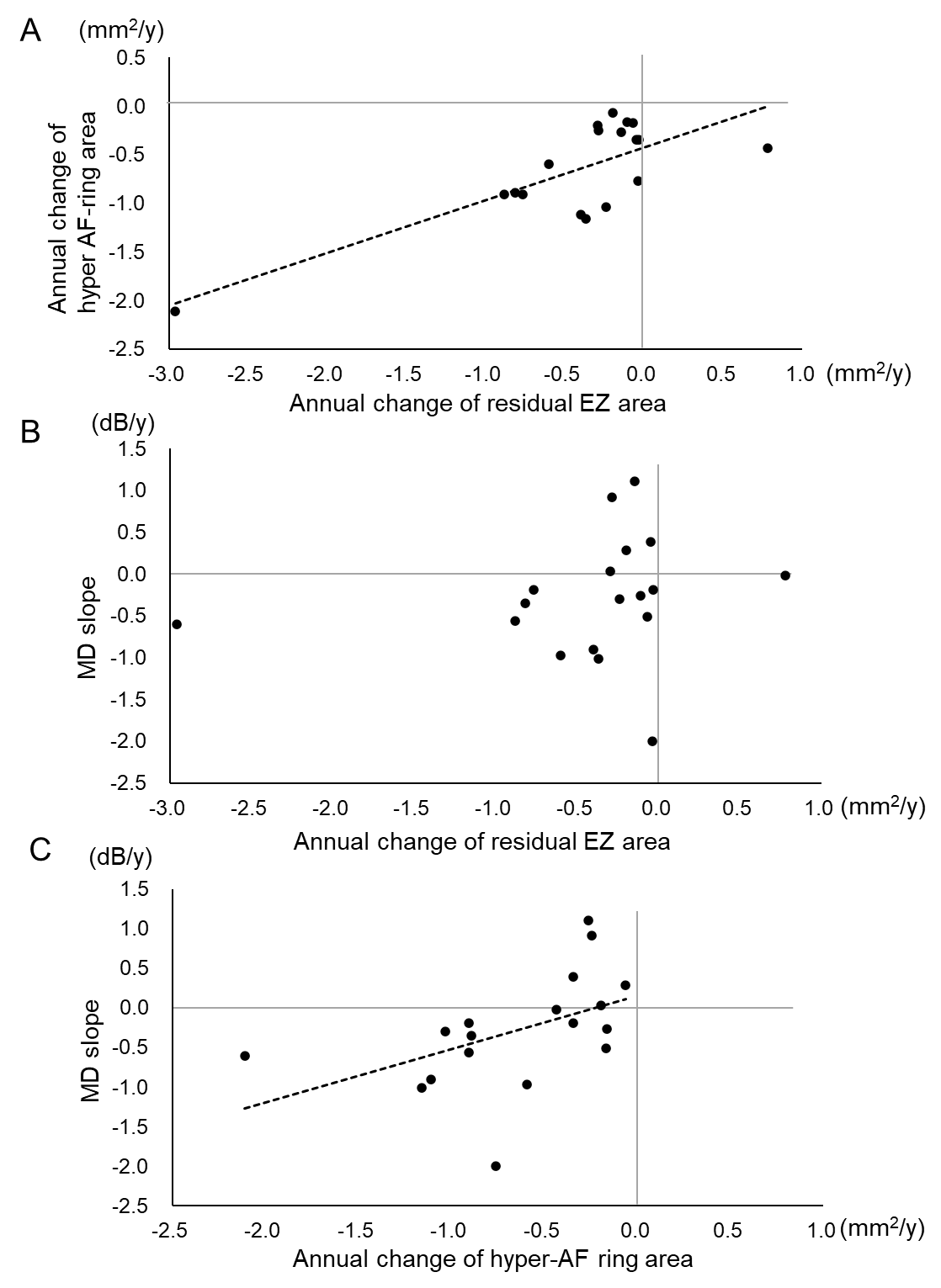


**Supplementary Figure S3. Correlations between annual change rates evaluated by different parameters in eyes with the causative *EYS* gene.** **(A)** The annual decrease rates of the residual EZ and hyper-AF areas are positively correlated (*P* = 0.0002, r = 0.78). **(B)** The annual change rate of the residual EZ area does not display a significant correlation with the MD slope (*P* = 0.49, r = 0.03). **(C)** Faster annual reduction rates of the hyper-AF ring area are significantly correlated with a steeper MD slope (*P* = 0.045, r = 0.48). The dotted lines in **(A)** and **(C)** denote the approximate straight line using linear regression analysis with the least-squares method.
